# Delivery of Intraocular Triamcinolone Acetonide in the Treatment of Macular Edema

**DOI:** 10.3390/pharmaceutics4010230

**Published:** 2012-03-15

**Authors:** Aaron Pickrell, Alon Harris, Sandra Ngo, Annahita Amireskandari, Erin Stewart, Brent Siesky

**Affiliations:** Eugene and Marilyn Glick Eye Institute, Indiana University School of Medicine, 1160 W. Michigan Street, Room 205Q, Indianapolis, IN 46202, USA; Email: pickrellaaron@gmail.com (A.P.); sango@iupui.edu (S.N.); aamiresk@gmail.com (A.A.); ehetzel@iupui.edu (E.S.); bsiesky@indiana.edu (B.S.)

**Keywords:** macular edema, intravitreal injection, sub-Tenon’s injection, corticosteroids, triamcinolone

## Abstract

Macular edema (ME) is one of the eventual outcomes of various intraocular and systemic pathologies. The pathogenesis for ME is not yet entirely understood; however, some of the common risk factors for its development have been identified. While this investigation will not discuss the numerous etiologies of ME in detail, it appraises the two most widely studied delivery modalities of intraocular corticosteroids in the treatment of ME—intravitreal injection (IVI) and sub-Tenon’s infusion (STI). A thorough review of the medical literature was conducted to identify the efficacy and safety of IVI and STI, specifically for the administration of triamcinolone acetonide (TA), in the setting of ME in an attempt to elucidate a preferred steroid delivery modality for treatment of ME.

## 1. Introduction

Macular edema (ME) is one potential outcome of various intraocular and systemic pathologies, such as diabetic retinopathy, retinal vein occlusion, and operative complications. Though the pathogenesis for ME is not yet entirely understood and highly dependent on the etiology of ocular injury, common risk factors for its development have been identified, including fluctuations in glucose level and retinal hypoxia, as seen in diabetic macular edema. Such insults are thought to cause a hypoxia-induced upregulation of VEGF and recruitment of inflammatory mediators to the retina, which allows fluid to collect behind the macula of the eye and leads to central visual distortion and potential vision loss if left untreated.

Given the variable nature of this process, current treatment and management of ME is a significant challenge to any practicing ophthalmologist. In general, the main goals of ME therapy are to: (1) reduce inflammation; (2) reduce VEGF production; and (3) reduce blood-retinal barrier (BRB) breakdown. Decreasing the inflammatory response and restoring tight junctions are key elements in restoring the healthy anatomy of the macula and vision [1]. This is most apparent in the cases of chronic and persistent ME, in which stepwise approaches to care must be considered. Though various treatment options are now available to achieve these goals of therapy, the use of corticosteroids has continued to be a mainstay in the management of ME.

Treatment with corticosteroids has been shown to be effective in reducing ME associated with diabetic retinopathy, vein occlusions, and other pathologies by inhibiting the formation of prostaglandins, which are potent regulators of inflammatory mediation. Additionally, corticosteroids contain certain anti-angiogenic properties that provide further benefit to patients suffering from ME [2]. Various routes of corticosteroid administration exist in the treatment of ME, including topical application, oral intake, and intraocular injection or infusion of steroids. However, oftentimes in clinical practice, intraocular injections and infusions have proven to be more efficacious than topical or systemic steroids in the management of certain ocular pathologies, of which ME is included. This difference in efficacy is attributed to the fact that corticosteroids are more likely to achieve therapeutic concentrations and their effects are sustainable for a greater duration of time when administered intraocularly [3]. Therefore, given the widespread use of these injections and infusions in patients with ME, we will conduct a thorough review of the two most extensively studied intraocular steroid delivery modalities—intravitreal injection and sub-Tenon’s infusions—and appraise the efficacy and safety of each in an attempt to better understand factors affecting the treatment of ME and reveal a preferred steroid delivery method in the management of this process.

## 2. Barriers Affecting Drug Delivery and Metabolism

Before discussing the different methods of intraocular steroid delivery, it is important to understand the three types of barriers that exist within the eye: static, dynamic, and metabolic. These barriers can affect the physical delivery of a drug, the efficacy of a drug once inside the eye, and its metabolism and clearance.

### 2.1. Static Barriers

Static barriers are those that provide a physical impediment to foreign substances. The static barriers found within the eye are composed of different segments—the cornea, sclera, retina, and the blood-retina barriers. *Ex-vivo* studies have been conducted extensively with respect to factors pertinent to the permeability of scleral tissue. Such influencing factors are molecular weight/molecular radius, lipophilicty, and charge. Permeability of scleral tissue for large and small molecules is low and high, respectively [4,5,6,7]. Interestingly, molecular radius has a more profound impact on permeability than molecular weight. In studies conducted on rabbits, globular proteins of the same molecular weight as linear dextrans proved to be more permeable [8]. Further research in this area has proven that as molecular radius increases there is an exponential decrease in permeability. These effects could be explained by the structure of the sclera. Scleral tissue is composed of collagen and elastin fibers arranged in a matrix array. This matrix allows for the presence of pores that may be of variable size in regional areas of the sclera (*i.e.*, looser fiber matrix in the posterior sclera compared to anterior) [9,10]. Permeability of scleral tissue decreases as the lipophilicty increases which is thought to be the result of proteoglycans found in the tissue. These proteoglycans allow easier passage of hydrophilic compounds when compared to lipophilic compounds [11]. Another variable to take into account would be with regards to charged particles, where negatively charged particles are more permeable than positively charged particles [12,13]. Positively charged particles are thought to form bonds with the negatively charged proteoglycans found in scleral tissue [14]. 

Studies involving Bruch’s membrane-choroid complex (BC complex) and retinal pigment epithelium (RPE) have not been as extensively investigated as the sclera [15]. Over the course of many different studies the conclusions with respect to changes in permeability have been identified. The BC complex behaves much like the sclera in regards to molecular radius, lipophilicity, and charged molecules [12,16]. RPE behaves nearly identical to sclera in reference to molecular radius. However, RPE behaves differently with regards to lipophilicty, where permeability increases as solute lipid solubility increases [17].

### 2.2. Dynamic Barriers

While static barriers and their permeability have been studied primarily ex-vivo, dynamic barriers can only be studied *in-vivo*. The properties that constitute dynamic barriers include: clearance of drugs through blood vessels and lymphatic flow, bulk fluid flow, and active transport within the RPE. Two experiments, done in parallel with rabbits, were conducted to determine which mechanism of clearance most extensively limits drug delivery via a transscleral modality. They first utilized cryotherapy to effectively destroy RPE and choroidal capillaries. The authors report that there was very little change in penetration of STI-TA post cryotherapy. In the second experiment a conjunctival/episcleral “window” was incised thus eliminating the blood vessels and lymphatics within these tissues. After formation of the window, placement of STI-TA was performed and significant penetration of the drug was observed. These results indicate that choroidal elimination of transscleral delivered drugs is minimal when compared to conjunctival/episcleral clearance [6,18,19]. 

Clearance studies have given rise to a better understanding of dynamic barriers while other areas such as bulk fluid flow, in the form of uveoscleral outflow, are still limited. While an unorthodox pathway for aqueous humor fluid flow in the eye, uveoscleral outflow may have a profound effect on drug delivery by trapping therapeutic agents in its convective flow of aqueous humor [20]. In addition, RPE transporters may have the added effect of eliminating therapeutic agents lowering drug bioavailablity. The presence of efflux pumps in the form of P-glycoprotien (P-gp) and multidrug-resistance associated proteins (MRPs) may direct drugs away from the retina and promote their clearance via the choroidal circulation [6]. 

### 2.3. Metabolic Barriers

Inherent metabolic enzyme systems are responsible for the eye’s protection against foreign molecules. While the primary sites of production of these enzymes have been examined, the study of these enzymes and their effects on pharmacokinetics of therapeutic agents is limited [21]. The two most widely studied metabolic enzymes within the eye are the cytochrome P-450 enzyme and lysosomal enzymes [6]. These enzymes may have a very important role in ocular drug delivery; however, more research is needed to understand their function in order to improve results. 

## 3. Intravitreal Injection of Corticosteroids to the Posterior Pole

The currently utilized practice of intravitreal injection (IVI) arose out of blood-ocular barrier research in the 1970s. This investigation initiated the concept of delivering corticosteroids to the vitreal cavity as a modality for treating intraocular inflammation [2,22]. Corticosteroids have been utilized for their anti-inflammatory properties by hindering mediator response and reducing cytokine production. However, the angiostatic and antipermeability characteristics of corticosteroids are the focus of more contemporary research studies for treatment of posterior segment diseases, including ME [23]. 

**Figure 1:**
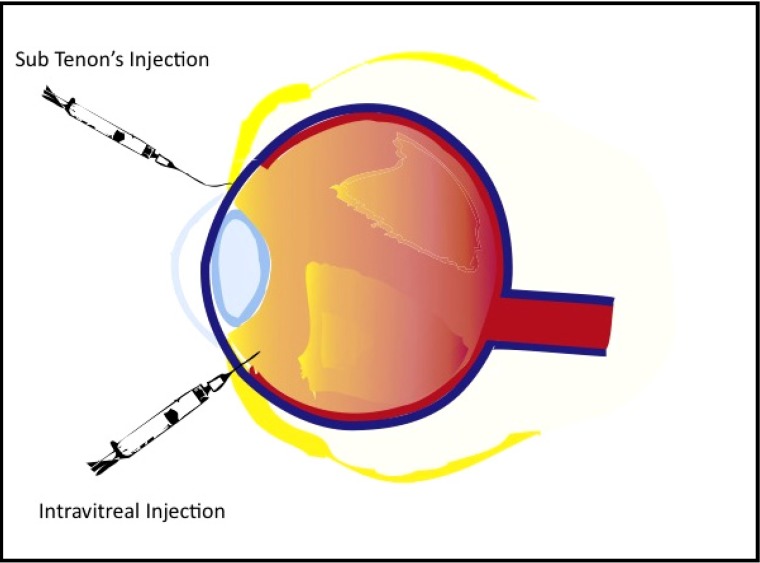
Diagram illustrating the two discussed routes of triamcinolone acetonide (TA) administration, sub-Tenon’s infusion (STI) and intravitreal injection (IVI). STI placement is between Tenon’s capsule and the sclerotic coat of the eye. This is accomplished by making a small incision and placing a blunt curved cannula towards the posterior pole of the eye and infusing slowly. IVI is placed 3 to 4 mm posterior to the limbus and in the inferotemporal region.

IVI is usually an in-office procedure performed using topical anesthesia. A survey of retinal physicians from 2006 reports that a majority of practitioners use topical anesthesia (66.6%) in contrast to subconjunctival anesthesia (33.3%) during this procedure [24]. Also, when performing IVI, proper aseptic technique should be executed along with the application of povidine-iodine solutions to decrease the risk for contamination by normal conjunctival flora. IVI technique employs the use of a compass to measure 3 to 4mm posterior to the limbus in order to identify the region of the pars plana. Through this region using a 30 to 32 gauge needle, therapeutic agents can be safely introduced into the vitreal chamber. The agent for IVI is normally injected slowly in the infero-temporal quadrant in order to prevent interference with the patient’s visual field [2]. (See Figure 1)

The benefits of IVI consist of sustained and sufficient release of the drug to the posterior segment of the eye while diminishing potential side effects of corticosteroids by circumventing the blood-ocular barrier. This drug delivery modality may also reduce the issue of noncompliance in patients [23]. However, there are also complications that have been associated with this procedure; a known consequence of electing to perform IVI is the patient’s associated risk of developing endophthalmitis. A report from the DRCRnet and SCORE trials provided a detailed procedure for the prevention of the development of endophthalmitis following IVI without the use of prescribing prior antibiotics. They reported an incidence of endophthalmitis of 0.05% (95% confidence interval, 0.001%–0.277%), with total IVI injections of n = 2009 [25]. 

### 3.1. Triamcinolone Acetonide

Triamcinolone acetonide (TA), specifically intravitreally-injected TA (IV-TA), has been extensively studied over the last decade as it is a common corticosteroid used in IVI for the treatment of ME. Steroids are effective in the treatment of intraocular edema because inflammation often accompanies and incites abnormal growth of intraocular cells. Furthermore, steroids help protect from fluid accumulation in the macula region caused by capillary defects in the blood-retina barrier. TA is one of the few corticosteroids available in a crystalline form, which allows for long-lasting concentrations in the vitreal chamber to be achieved without the addition of a vehicle to improve sustainability [3,26,27]. 

### 3.2. Efficacy of IV-TA

While variations in the primary outcome measurements of different clinical trials do exist with regard to treatment with intravitreal-TA (IV-TA), they ultimately point to a common assessment of its efficacy. Most outcomes were measured on basis of ≥5-letter increase in best-corrected visual acuity (BCVA) and changes in central macular thickness (CMT). A two-year, double-blinded and randomized clinical trial reported that with treatment of 0.1 mL TA (40 mg/mL [Kentacort 40 Bristol-Meyers Pharmaceuticals]), 19 of 34 IV-TA patients compared to 9 of 35 placebo patients saw an improvement of ≥5 letters (*P* = 0.006). In this same study, foveal thickness was reduced by up to 59 µm in the IV-TA group as compared to the placebo group [28]. Another clinical trial with nearly identical treatment protocol reported 18 of 33 IV-TA patients compared to 5 of 32 placebo patients saw an improvement of ≥5 letters (*P* < 0.001). Additionally, macular thickness was significantly reduced by at least 1 grade in 25 of 33 IV-TA patients versus 5 of 32 placebo patients (*P* < 0.0001) [29]. In a 2011 study involving IV-TA and laser treatment, Gillies *et al.* reported that an improvement in BCVA of 10 letters or more recorded in logarithm of the minimal angle of resolution (logMAR) units was found in 15 of 42 eyes treated with TA prior to laser treatment compared to 7 of 42 eyes with placebo over a 24-month period (*P* = 0.047). Odds of improving by 10 letters or more were 2.79 times greater (Confidence interval 95%, 1.01–7.67) before laser therapy than in eyes treated exclusively with laser. While the IV-TA group showed a decrease in CMT, there was no statistically significant difference in CMT between the two groups [30]. 

Whereas the aforementioned clinical trials used a dosage of 4 mg TA, other studies have investigated the differences in outcome seen when a range of doses is administered. Evaluating relatively small dosage amounts, one investigation reported the effects of IV-TA at 1 mg, 2 mg, and 4 mg, where n = 13, 17, 12 respectively, and in this study, all three dosage groups increased by 8 or 9 Early Treatment Diabetic Retinopathy Study (ETDRS) letters by 4 weeks. Interestingly, the 1 mg and 2 mg groups showed no remission at 24 weeks, while the 4 mg group showed a slight remission; however, in the course of the six-month study, there was no statistically significant difference among the three dosage groups. Baseline studies of CMT were similar among all three dosage groups, and all three improved by 4 weeks post-injection. Standardized-CMT (SCMT) was calculated for all three groups according to the equation outlined by Chan and Duker, 2005. The authors explained that the SMCT values for the 4 mg dosage group were significantly worse than the 1 mg at all time points and worse than the 2 mg group at the 12- and 24-week follow-up; this lack of correlation between macular thickness and improvement in VA had been identified in previous studies [31,32]. In a study regarding the duration of effects of IV-TA dosages ranging from 20 to 25 mg, the authors reported an increase from a baseline of 0.93 ± 0.28 logMAR units to 0.79 ± 0.34 logMAR units at 1 month post-injection [26], and the gain in VA achieved a plateau-like effect between 1 to 7 months post-injection, returning to baseline between 8 and 9 months. However, it should be noted that there are several limitations described by the author of this study that hindered the gain of other significant conclusions on the effects of these dosages [30]. 

### 3.3. Drawbacks of IVTA

Various studies highlighting the adverse effects associated with IV-TA use, including endophthalmitis, have also been performed. Maia M., *et al.* conducted a retrospective analysis in n = 471 patients receiving IVIs of Kenalog (KE) (Kenalog, Bristol-Myers Squibb, Princeton, NJ, USA) and preservative-free triamcinalone acetonide (PFTA) in which they reported that, in 646 IVIs of steroids, 12 eyes developed non-infectious endophthalmitis. Of these 12 eyes, five patients received injections of KE (n = 69, 7.3%) and seven received injections of PFTA (n = 577, 1.2%), and the results of this study were statistically significant (*P* = 0.005) for differences between the two preparations [33]. Additionally, a prior study by Nelson *et al.* documented similar findings as well as case reports of sterile endophthalmitis [34]. Interestingly, however, inflammation does not seem to be isolated solely to the intraocular use of TA; such events have been also documented following the intra-articular injection of TA as well [35]. 

Other concerns regarding IV-TA include visual disturbances, elevation of intraocular pressure (IOP), and the risk of quickened cataract progression [3,36,37,38,39]. Patients reported, in a 2011 clinical study by Charalampidou *et al.*, that they experienced flashing lights and floaters in their vision immediately following and up to 2 weeks post-procedure. These phenomena may be attributed to viscous deposits that are observed to accumulate on the surface of the eye and the transient increase in IOP immediately following injection, as elevated IOP after corticosteroid use has been a known complication for some time [36,40,41]. Jonas *et al.* investigated the implications of the IOP response to IV-TAs and found that IOP was increased significantly from 15.4 mmHg to a mean maximum of 23.34 mmHg postoperatively (*P* < 0.001). Post-injection increases in IOP to greater than 21 mmHg were found to be statistically independent of sex, refractive error, presence of diabetes, and indication for the injection (age-related macular degeneration versus DME) [37]. Inatani *et al.* examined the various risk factors for the elevation of IOP in conjunction with IVI-TA use. The author’s used a Cox proportional hazards regression and found that IOP elevation after IVI-TA is independent of age and baseline IOP but found significant risk associated with higher dosage administration (*P* = 0.013) [42]. With respect to IV-TA and laser treatment, Gillies *et al.* explained that 27 of 42 IV-TA plus laser patients required management for elevated IOP as compared to 10 of 42 laser only eyes (*P* < 0.001) [30].

In addition to the laser treatment report, Gillies *et al.* have published an analysis of the effects of IV-TA on cataract development and progression. Over a three-year study, 2 of 25 sham injection patients together with 15 of 27 IV-TA patients underwent cataract surgery. All eyes showed ≥2 grade posterior subcapsular cataract (PSC) with significant visual impairment. They reported that in the initial steroid-treated group, cataract development took more than 12 months, and a further breakdown identified that 10 of 15 eyes that had three injections had progression of PSC, while only 5 of 12 eyes with less than three injections progressed (*P* = 0.009) [38]. The authors found reproducible results that demonstrated the association of PSC progression with increased IOP ≥ 5 mmHg from baseline at any time [39]. In a similar study by Gillies *et al.*, cataract progression by 2 or more Age Related Eye Disease Study (AREDS) grades was found in 18 of 28 and 3 of 27 eyes that were phakic at baseline in the IV-TA plus laser group compared to the laser only group, respectively (*P* < 0.001) [30].

## 4. Sub-Tenon’s Infusion of Corticosteroids to the Posterior Pole

There are currently a few different methods of administering corticosteroids through sub-Tenon’s space discussed in the literature. Previously, it was common practice to perform sub-Tenon’s injections, using a sharp needle to inject drugs into the space beneath Tenon’s capsule. However, this carried the risk blepharoptosis and globe perforation [43]. Additionally, a bolus injection of drug may be less effective than a more gradual infusion as the sclera can become saturated, limiting the amount it is able to absorb at a given time [6,7]. More recently, most clinicians choosing the sub-Tenon’s route for steroid delivery employ a blunt, curved cannula for infusion rather than injection with a sharp needle. This long cannula is meant to reach behind the posterior globe to allow the steroid to infuse into the retrobulbar space, while reducing the risk of complications associated with needle use [43]. This method is often called trans-Tenon’s or sub-Tenon’s infusion (STI) (see Figure 1). Though this modality is used in clinical practice, it has been cited as a less efficient method of drug delivery to the retina than IVI due to the various barriers encountered between the sclera and the posterior pole. These barriers, as explained above, have been classified into three types: static, dynamic, and metabolic [6].

### 4.1. Efficacy of STI-TA

A few studies have been conducted which evaluate the efficacy of STI corticosteroid delivery in the management of ME. Of note, one particular study reported results for the outcomes after STI steroid administration, which were comparable to those found in the aforementioned literature on IV-TA in eyes with ME. This study by Tomoyo K *et al.* examining the efficacy of trans-Tenon’s retrobulbar TA injections showed the final Snellen BCVA improved by two or more lines in 9 eyes and remained unchanged in 11 eyes. The authors indicate at the final follow up ME resolved or improved in 85% of the cases included [44]. Additionally, in a yearlong analysis comparing IVI and STI, researchers evaluated both modalities using CMT, BCVA, IOP, and cataract progression as parameters. A significant (*P* < 0.01) reduction in CMT for IVI groups at weeks 2, 4, 8, 12, 24 post-injection was observed when compared to this type of transscleral injection. Values for IVI and STI were then independently compared to baseline evaluations, and only IVI presented significant reduction in CMT from baseline, while changes seen after STI were not found to be significant at any time point. This same analysis showed significant (*P* < 0.01) logMAR BCVA improvements in the IVI group versus those in the STI group, and when individually compared to baseline measurements, STI of corticosteroids failed to show significant improvement in contrast to IVI [45]. These findings were comparable to those reported in a similar study by Cardillo *et al.*, which noted an elevation in IOP for both groups without any adverse events [46].

### 4.2. Drawbacks of STI-TA

The risk profile associated with STI, specifically in the area of IOP elevation, seems to be less than that observed for IVI drug delivery. In previous studies, IOP elevation following IV-TA treatment has been reported in roughly 20% to 80% of patients [37,47,48]. The aforementioned study by Inatani *et al.* examined the various risk factors for IOP elevation following STI-TA injection. The authors identified statistically significant risk factors for IOP elevation after STI-TA to be younger age, higher dose administration and higher baseline IOP (*P* = 0.003, *P* = 0.0006, *P* = 0.0003 respectively) [42]. Muller *et al.* employed sub-Tenon’s injections with five-eighths inch, 25-gauge needle, rather than the more commonly used blunt cannula, and reported that the sub-Tenon’s injection modality of corticosteroid delivery, even with various different steroid medications administered, seemed to be less disposed to produce significant intraocular hypertension [49]. Another study by Cardillo *et al.*, elicited no increase in IOP to >25 mmHg treatment of DME with STI-TA [46]. Although the exact mechanism behind IOP elevation following ocular corticosteroid administration is not completely appreciated, it is thought to be derived from an increased resistance to aqueous humor outflow within the eye, and understanding the reason behind such a disparity between IVI and STI of corticosteroids regarding IOP elevation may be significant for comprehending their differences in efficacy. With regards to cataract progression Bonini-Fihlo *et al.* report no incidence of cataract progression within their study population. However, the authors do discuss the need for further evaluation and follow up as the limited time period of this study may not show progression. One must also consider that STI is an inherently more difficult procedure to perform. Shimura *et al.* investigated the effects of drug reflux (back flow of TA through the sub-tenon’s incision) on foveal thickness (FT) and elevations of IOP in patients with DME with a foveal thickness > 400 µm and a VA less than logMAR 0.3. Out of 128 patients in the study, 10 experienced drug reflux. Comparing the drug reflux positive group with the reflux negative group, FT was higher at all time points for the reflux (+) group (*P* = 0.004). In addition the authors noted a increase in IOP to >25 mmHg in 4 out of 10 eyes in the reflux (+) group in comparison to 3 out of 188 eyes in the reflux (-) group [50]. See Table 1 for a summary of the advantages and disadvantages of IVI and STI.

**Table 1:** Advantages and disadvantages of IVI and STI use.

IVI		STI	
Pro	Cons	Pro	Cons
Effectively reduces macular thickness and improves BCVA	Faster development of cataracts	Small improvement in BCVA and macular thickness	Technically more difficult to perform Correctly (*i.e.*, risk of reflux)
Increased drug bioavailability	Increased risk of endophthalmitis	Less risk of cataract development	Many barriers that ultimately reduce drug bioavailablity
	Increased risk of elevated IOP and secondary glaucoma	Less risk of secondary intraocular hypertension (if no reflux during procedure)	

## 5. Conclusions

ME can develop as a result of a variety of ocular and systemic conditions including diabetes, retinal vein occlusion, and uveitis. The management of ME is varied and largely dictated by its etiology; however, given their potent anti-inflammatory properties, the use of corticosteroids has been exceedingly popular in treating this process, especially when administered intraocularly. We reviewed the two most widely studied forms of intraocular steroid delivery—intravitreal injection and sub-Tenon’s infusion. Currently, there seems to be a preference in treatment of ME with IVI over STI as literature suggests significant improvement in BCVA and reduction in CMT has been demonstrated in patients treated with IVI, whereas such improvements have not typically been seen with STI. Based on these findings, it is assumed that direct injection of corticosteroids into the vitreous allows for more effective and sustained release of the drug to the posterior segment of the eye than that which can be achieved when corticosteroids are infused beneath Tenon’s capsule; this difference in efficacy has been attributed to the various barriers (static, dynamic, metabolic) that exist between the sclera and posterior pole and are encountered by a drug that is administered via STI. Given the implications of these barriers in the management and outcome of treatment, modification of the corticosteroid delivery vessel may offer some potential for increasing the effectiveness of STI. Indeed, the efforts of Boddu *et al.* have recently investigated the use of nanoparticulate gel formation for the delivery of steroids via a transscleral approach [51]; however, though this research appears promising, it may be some time before clinicians witness this preparation in human clinical trials. 

A specific focus on the efficacy and safety of triamcinalone-acetonide (TA) was also included in this review, as it is the most commonly used intraocular steroid in the treatment of ME, and several studies report benefit from treatment with this corticosteroid. In the current literature, IV-TA has been associated with a statistically significant improvement in BCVA as well as a significant reduction in macular thickness. However, there also seems to be an increased risk for developing endophthalmitis with the use of IV-TA, which emphasizes the importance of using proper aseptic technique to reduce the likelihood of this event. Also, as with use of any intraocular steroid, an increased risk of developing elevated IOP, secondary glaucoma, and cataracts has been documented with use of IV-TA, and it is important to note that STI of TA seems to carry a lower risk for the development of ocular hypertension. As ocular barriers are known to hinder drug efficacy, they may also play a role in this observation by limiting the adverse effects of intraocular steroid treatment. 

We can conclude that certain aspects of IV-TA and STI-TA treatment still need to be explored more thoroughly in order to optimize the management of ME. For instance, one issue that needs further clarification is that of ideal steroid dosage. Many studies have evaluated treatment with a 4 mg dose of TA, while others have even assessed the use of both higher and lower doses of TA. A comprehensive, retrospective meta-analysis comparing these outcomes may elucidate the most efficacious corticosteroid dosage for treating ME. Further research investigating barriers to drug delivery is also needed. A comprehensive understanding of the pharmacokinetics attributed by each barrier could increase drug bioavailability and ultimately improve treatment outcomes. Also, the applications of IV-TA and STI-TA use in combination with other treatment modalities such as photodynamic therapy, as well as the possible combination of these two injection techniques used in tandem with different therapeutic agents (*i.e.*, anti VEGF factors and TA), will be of further interest regarding the future treatment of ME as well as in the management of other intraocular inflammatory processes. 
